# The Influence of Cement Layer Thickness on the Stress State of Metal Inlay Restorations—Photoelastic Analysis

**DOI:** 10.3390/ma14030599

**Published:** 2021-01-28

**Authors:** Grzegorz Sokolowski, Michal Krasowski, Agata Szczesio-Wlodarczyk, Bartlomiej Konieczny, Jerzy Sokolowski, Kinga Bociong

**Affiliations:** 1 Department of Prosthetics, Medical University of Lodz, 92-213 Lodz, Poland; grzegorz.sokolowski@umed.lodz.pl; 2 University Laboratory of Materials Research, Medical University of Lodz, 92-213 Lodz, Poland; michal.krasowski@umed.lodz.pl (M.K.); bartlomiej.konieczny@umed.lodz.pl (B.K.); 3 Department of General Dentistry, Medical University of Lodz, 92-213 Lodz, Poland; jerzy.sokolowski@umed.lodz.pl (J.S.); kinga.bociong@umed.lodz.pl (K.B.)

**Keywords:** stress analysis, dental restoration repair, prosthetic dentistry, water absorption

## Abstract

The successful restoration of teeth requires a good connection between the inlay and natural tissue. A strong bond may improve retention and reinforce tooth structure. The purpose of this study was to evaluate the influence of cement layer thickness on contraction stress generated during photopolymerization, and to determine the changes in stress state of the cement occurring during aging in water (over 84 days). Two cements were used: resin composite cement (NX3) and self-adhesive resin cement (Maxcem Elite Chroma). A cylindrical sample made of CuZn alloy was used to imitate the inlay. The stress state was measured by photoelastic analysis. The contraction stress of the inlay restoration was calculated for cement layer thicknesses of 25 µm, 100 µm, 200 µm, and 400 µm. For both tested materials, the lowest contraction stress was observed for the thinnest layer (25 µm), and this increased with thickness. Following water immersion, a significant reduction in contraction stress was observed due to hygroscopic expansion. Applying a thin layer (approximately 25 µm) of composite and self-adhesive resin cements resulted in high levels of expansion stresses (over −6 MPa) after water aging.

## 1. Introduction

Damaged teeth which are not able to support basic restorations are typically repaired using indirect restorations including inlays and onlays [1]. Onlays are applied differently to inlays in molars with two or even three cusps missing. Unlike crowns, inlays or onlays cover only the part of the tooth; however, metal-ceramic inlays are better at preserving natural tissue and tooth vitality, and reducing postoperative sensitivity than crowns, and they are less invasive. In addition, a gravimetric analysis of removed tooth structure found less tooth reduction associated with ceramic veneers and onlays compared to all-ceramic crowns [2,3]. Metal-reinforced systems are very often chosen to manufacture posterior fixed partial dentures [4]. Such metal-ceramic inlay-retained fixed partial dentures (IRFPDs) offer a number of advantages such as greater tooth structure preservation, and a lower risk of gingival irritation and pulp vitality. The method of tooth preparation for inlays or IRFPDs is similar to that used for indirect preparation of class II cavities [5,6,7,8].

The durability of indirect restorations depends upon that of the strength of the bond between the tooth and the restoration [9]. Hence, to achieve strong, durable adhesion to dental hard tissues, inlays are cemented with glass ionomer, resin-modified glass ionomer cements, and resin composite cements [10,11]. For decades, zinc phosphate cement has been the most commonly-used material for cementing, although resin luting agents have been proposed as alternatives [12]. It has been reported that resin cements show better bond strength to dentin compared with zinc-phosphate or glass ionomer cement [13]. Three types of resin cements exist based on the adhesion procedure: resin composite cement, adhesive resin cement, and self-adhesive resin cement [14].

As resin composite cements require multi-step application, which is time-consuming and susceptible to manipulation errors that may affect bond strength [15,16], self-adhesive resin cements were designed. This type of material bonds directly to the tooth tissue without any surface pre-treatment (priming) or conditioning [17,18]. However, following cementation resin shrinkage caused by polymerization can occur, and this may affect the integrity of the interface between the resin cement and the tooth structure [13]. The degree of contraction stress exerted by the resin cement depends on the material; however, shrinkage stress may be partially relieved, and in some cases, the tooth tissue can be compressed by hydroscopic expansion of the material caused by water uptake [19].

However, there is little understanding of how metal inlays and luting agents affect stress distribution in the tooth tissue and the changes that occur during their aging in water. Especially, if the reconstruction procedure has to be repeated and the possibility of a perfect fit is limited. In such cases, there is a thicker layer of cement [20]. Composite inlays are becoming more popular, mainly due to their aesthetic value, and have been used in most studies on this area [21]; however, a metal inlay was chosen for the present study to negate the influence of water absorption (associated with composite materials) and clarify the effect of absorption on the stresses occurring at the interface between the cement layer and the tooth tissue (epoxy resin).

The aim of this study was to evaluate the influence of cement layer thickness on the contraction stress generated during photopolymerization. It also evaluated the changes in stress state occurring during water aging of resin cements using the photoelastic method.

The following null hypotheses were stated:

1. The change in cement volume does not affect shrinkage stress.
2. Water absorption does not affect the state of stress.

## 2. Materials and Methods

The composition of investigated resin cements was presented in Table 1.

### 2.1. Photoelastic Study—The Dependence of the Cement Layer Thickness on Shrinkage Stress

Transparent and photosensitive plates made of epoxy resin (Epidian 53, Organika-Sarzyna SA, Nowa Sarzyna, Poland) with a Young’s modulus similar to dentin were used to determine the contraction stress. Orifices 6 mm in diameter were sandblasted with 50 µm grain corundum (Cobra, Renfert, Hilzingen, Germany) to obtain higher micromechanical retention.

To achieve layers with uniform thickness, cylindrical samples (simplified inlays) were used. Cylindrical metal inlays with a height of 4 mm and diameters of 5.20, 5.60, 5.80, or 5.95 mm were prepared. Using such metal inlays, cement layers were obtained with the following values: 400 µm, 200 µm, 100 µm, and 25 µm. Our previous data indicates that zirconium, Co-Cr alloy, and CuZn alloy inlay restorations are known to have similar stress states [22]. Therefore, due to its ease of processing, CuZn alloy (type MM54, Huta Będzin, Będzin, Poland) was selected for inlay production. Metal inlays were sandblasted with 50 µm grain corundum and then cleaned in isopropyl alcohol (Organika) in the ultrasonic cleaner (EasyClean, Renfert GmbH, Hilzingen, Germany). Next, the bonding system (OptiBond^®^ XTR, Kerr) was applied on both the inlay and the surface of the plate and then polymerized with a light curing unit (Elipar S10, 3M ESPE, St Paul, MN, USA). The orifices were filled with resin composite cement or self-adhesive resin cement (for the test group, respectively) and selected inlays (with different dimensions) were inserted. Three samples were prepared for each tested group. The polymerization was performed according to the manufacturer’s instructions (Table 1). The light curing units had an output irradiance of 1450 mW/cm^2^, as stated by the manufacturer.

The stress acting at the interface around the inlays was determined indirectly by using the photoelastic method with a circular transmission polariscope (FL200, Gunt, Hamburg, Germany). The generated strains were visualized in photoelastic images, which were registered by a digital camera (Canon EOS 5D Mark II, Canon Inc., Tokyo, Japan). Stress and strain were analyzed two-dimensionally for stresses and three-dimensionally for deformations. The radial (σ_r_), circumferential (σ_θ_), and shrinkage stresses were calculated as described previously [23,24,25]. In total, 30 samples were tested.

### 2.2. Photoelastic Study—Influence of Water Absorption on Stress State

A 5.95 mm diameter inlay (25 µm cement thickness) was chosen as a sample with the lowest stress state, and a 5.60 mm inlay (200 µm cement thickness) as one with the highest stress state.

The epoxy resin plate with drilled and sandblasted orifices was placed in a container with distilled water and sealed in a laboratory incubator (CLW STD 115 STD, POL-EKO) at 37 °C for three months. This procedure allowed for full water absorption.

After three months, the soaked plates were removed from the incubator and their orifices were dried with compressed air and isopropyl alcohol. Samples were taken and the stress state was determined by the photoelastic method [22,23,24]. Three samples were prepared for each tested group. Pictures were taken of the samples; following this, the samples were placed in a container with water and sealed in a laboratory heater (CLW STD 115 STD, POL-EKO) at 37 °C. The stress state analysis was repeated after 1, 3, 4, 7, 10, 14, 21, 28, 56, and 84 days. In total 12, samples were tested.

## 3. Results

### 3.1. Photoelastic Study—Dependence of Cement Layer Thickness on Shrinkage Stress

**Figure 1:**
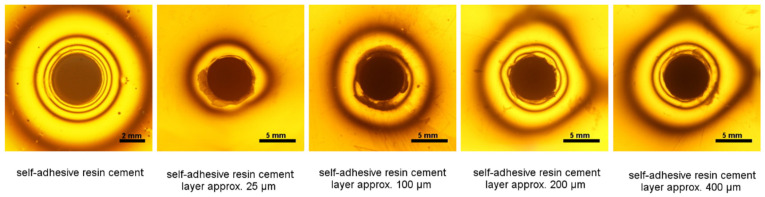
Isochromes in the epoxy plate around inlays with different thickness of self-adhesive resin cement (Maxcem Elite Chroma) layer (25 µm, 100 µm, 200 µm, and 400 µm).

**Figure 2:**
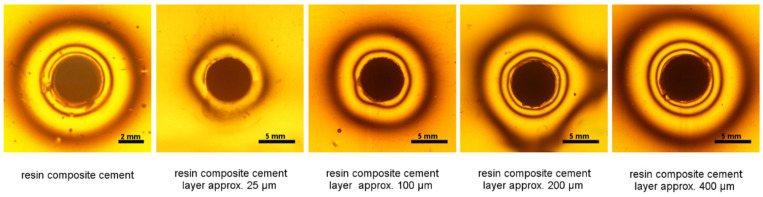
Isochromes in the epoxy plate around the resin composite cement and post-restoration with different thickness of cement (NX3) layer (25 µm, 100 µm, 200 µm, and 400 µm) around inlays.

The stresses generated during the polymerization of cement in the tested configuration, i.e., with metal inlays, are smaller than those generated by the material itself (Table 1 and Table 2). The lowest stresses were observed for the thinnest cement layer (approximately 25 µm). Increasing the cement layer from approximately 200 µm to approximately 400 µm did not significantly affect the observed shrinkage stress (Figure 1 and Figure 2).

### 3.2. Photoelastic Study—Influence of Water Absorption on Stress State

**Figure 3:**
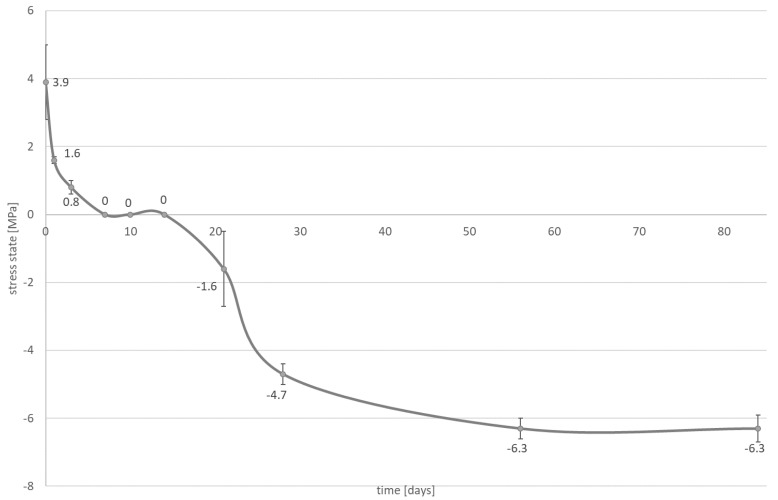
The influence of water absorption (84 days of water aging) on stress state (mean value and standard deviation) observed on bonded interface between epoxy resin plate and resin composite cement; the NX3 layer is approximately 25 µm.

**Figure 4:**
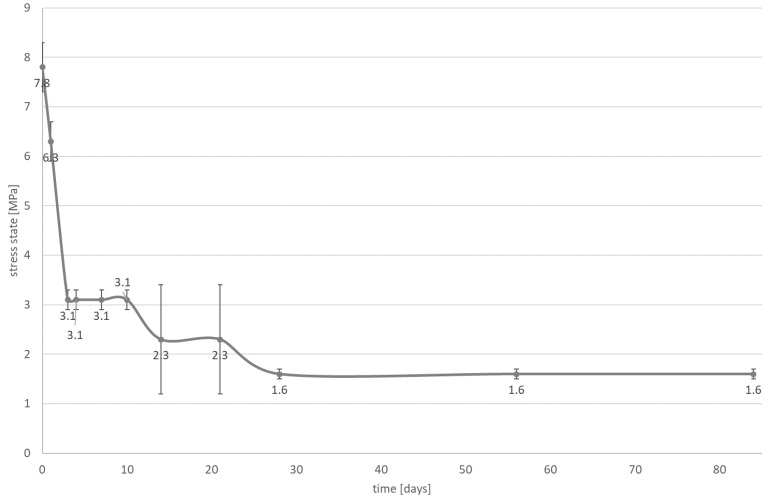
The influence of water absorption (84 days of water aging) on stress state (mean value and standard deviation) observed on bonded interface between epoxy resin plate and resin composite cement; the NX3 layer is approximately 200 µm.

**Figure 5:**
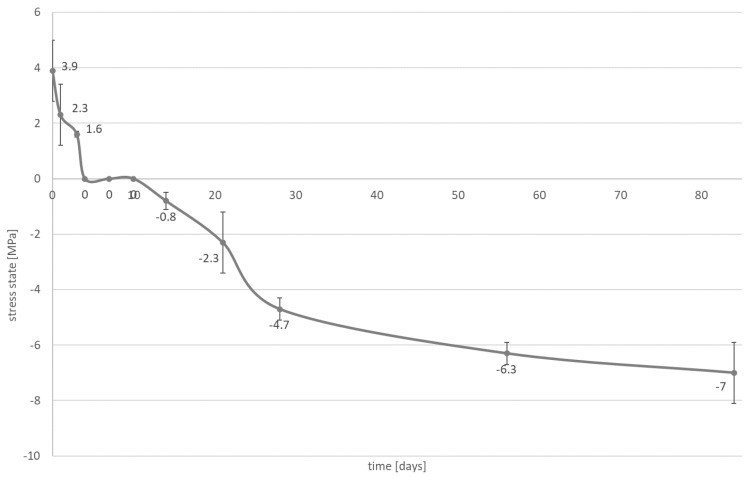
The influence of water absorption (84 days of water aging) on stress state (mean value and standard deviation) observed on bonded interface between epoxy resin plate and self-adhesive resin cement; the Maxcem Elite Chroma layer is approximately 25 µm.

**Figure 6:**
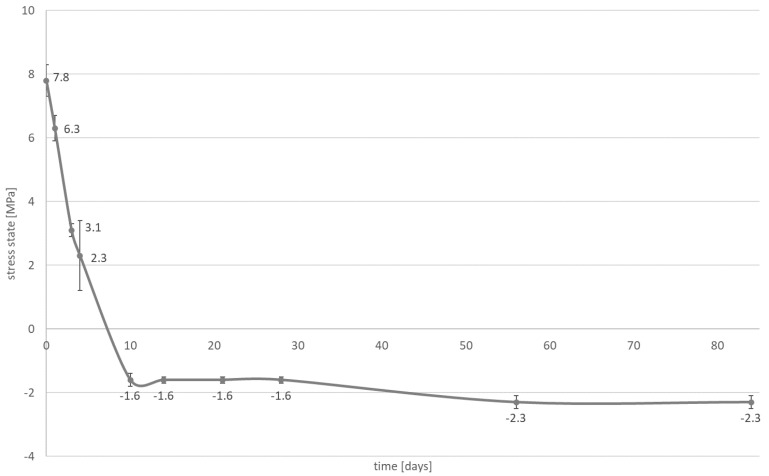
The influence of water absorption (84 days of water aging) on stress state (mean value and standard deviation) observed on bonded interface between epoxy resin plate and self-adhesive resin cement; the Maxcem Elite Chroma layer is approximately 200 µm.

A significant reduction in the contraction stress was observed, which could be attributed to the hygroscopic expansion of the cements (Figure 3, Figure 4, Figure 5 and Figure 6). However, the resin composite cement (NX3) with the 200 µm cement layer demonstrated no expansion stress associated with water aging (Figure 4). After 84 days water immersion, the self-adhesive resin cement with the 200 µm cement layer demonstrated lower stress than the cement with the 25 µm layer.

## 4. Discussion

All currently-available resin-based materials demonstrate material shrinkage on application due to polymerization [26,27,28]. Resin cements are similar to low-viscosity composites which exhibit a relatively high shrinkage (up to 6%) [29,30], and are generally applied as a thin layer. In addition, cavity preparation in prosthodontic restorations has a high C-factor, i.e., a low number of unbounded surfaces and high number of bonded surfaces [19,31]. These circumstances may generate sufficient stress resulting in debonding and formation of microleakage [32]. However, restorations are exposed to oral fluids, which may cause relief or even over-compensation of polymerization shrinkage [33].

Saliva is a more aggressive environment than water itself, with the resin demonstrating higher levels of sorption in saliva than in water alone. Additionally, the conditions of the oral cavity environment can accelerate the hydrolysis of the dental material [34], and saliva contamination has shown to be detrimental to adhesive bonding. Despite this, saliva contamination has not been found to influence the properties of composite materials with regard to their degree of conversion or microhardness [35].

Aging in water has been found to influence the stress state of resin cements [19]. The greatest water absorption has been observed for self-adhesive resin cement. This material was shown to induce water absorption, leading to the expansion of the polymer matrix. This can be explained by the fact that self-adhesive resin cement consists of resins (HEMA and GDM) which show one of the highest hydrophilicity among dental resin [19,36,37]. Resin composite cement demonstrates similar levels of absorption to composites because it does not contain adhesive monomers [19,24,25]. Studies have found that the composition of a material has the greatest impact on its hygroscopic expansion, plasticization, and resulting compensatory effect [38].

The first null hypothesis, which stated that the change in cement volume does not affect shrinkage stress, can be rejected. Our results indicate that contraction stresses increase with the thickness of the layer (Table 2 and Table 3). Similar observations have been reported in previous studies, which indicate that stress development increases with the volume of material with a constant bonded area [39]. A cement thickness greater than 400 µm does not appear to have any significant influence on the values of observed stress; such thicknesses are not used clinically anyway, as film thicknesses greater than 200 μm should be avoided due to a tendency to develop maximum stress zones [40,41]. Clinicians should strive to achieve the best possible match for indirect restorations, i.e., the marginal and internal fit of the indirect restoration should not exceed 50 µm for resin cements as specified by the ISO standard [42]. However, sometimes, it is not possible to achieve a perfect, evenly-distributed cement layer over the entire restoration interface; in such cases, 90 µm-thick layers are clinically acceptable, and higher values may exist in some places (pointwise) [43]. Even in laboratory conditions is difficult to obtain a cement layer of the same thickness (e.g., KoNroot Cem min. 49 vs. max. layer thickness 129 µm or Panavia F 2,0 min. 41 vs. max. layer thickness 80 µm) [44]. The presented study simulated clinical conditions where the fit of an indirect restoration was not perfect and the thickness of the cement could vary within the internal fit of the indirect restoration; such a situation may also result from the debonding of the inlay/onlay. This would result in the need to clean the connecting surface of the restoration and to re-prepare it with adhesive before re-cementing.

Two types of inlays were selected for further investigation: those with a cement thickness of 25 μm to give restorations that fit well clinically, and those with a cement thickness greater than 25 μm, which fit poorly. The second hypothesis can be rejected. It is observed that the stress state is affected by water absorption (Figure 3, Figure 4, Figure 5 and Figure 6). During the water aging test, it was found that for both tested materials, the use of a thinner cement layer resulted in a greater absolute stress change (Figure 3, Figure 4, Figure 5 and Figure 6). The samples with the thinner layer demonstrate small stresses resulting from shrinkage after polymerization (3.9 MPa for both cements); however, high expansion stress (over −6 MPa) is observed following water absorption (Figure 3 and Figure 5). In contrast, the thicker cement layer generated a higher (7.8 MPa) initial stress state, caused by shrinkage of the material during polymerization; however, a more than 50% reduction in stress state was noted after three days in water (Figure 4 and Figure 6). In addition, the system can be stabilized just after one month.

The samples of resin composite cement (NX3) stabilized at a level of 1.6 MPa (Figure 4), while those of self-adhesive cement (Maxcem Elite Chroma) stabilized at −2.3 MPa, i.e., hydroscopic expansion stress (Figure 6). Such shrinkage compensation caused by hygroscopic expansion can be attributed to the viscoelastic properties of composites. Water absorption increases relaxation through chemical degradation of the polymers (hydrolysis) and molecular mobility (plasticization effects) [45]. A thin layer of cement demonstrates greater restriction, resulting in the effects of hygroscopic expansion being transferred directly to the bonded materials, with significant changes being observed at the interface between the cement and the tissues (epoxy resin). In a larger volume of material, contraction stress compensation may occur and the ultimate stress state at the interface is smaller.

An appropriate cavity design and bonding agent may reduce gap formation. However, interfacial and/or marginal defects have been commonly observed under bonded indirect restorations in laboratory studies. Evidence of insufficient marginal sealing has also been shown in microleakage studies [46]. Hygroscopic expansion following water absorption relaxes the internal stresses of the resin restoration [19,24,25]. It can also reduce any gap formed due to polymerization shrinkage [47]. Consequently, the positive effect of water absorption on the marginal gap size depends on the thickness of the cement and composition of the luting materials. Our research indicates that a very thin cement layer (approximately 25 μm) of resin composite or self-adhesive resin cements may create a significant degree of expansion stress state. A high value of expansion stress may lead to failure of the tooth structure or ceramic crowns. It was shown that hygroscopic expansion of the resin modified glass ionomer (RMGIC) or compomer materials used for both core build-up or adhesive bonding, caused the failure of all-ceramic crowns [48]. However, the application of a cement layer measuring approximately 100–200 μm, particularly when using self-adhesive cements, may result in the development of expansion stress, which can seal the bond between cement and tooth. If cements characterized by lower water absorption are used, the initial level of stress will decrease.

The two cements were chosen to assess stress changes under conditions simulating clinical settings had significantly different levels of generated shrinkage stresses and water absorption [19,49]; however, despite these significant differences, the samples with the 200 µm-thick cement layer demonstrated relatively low stress values, due to water absorption. Hence, the described situation is clinically very favorable.

From the clinical point of view, our findings could improve the selection of an appropriate cement for indirect restorations: the candidate would demonstrate a good level of specific water absorption that would balance the contraction stresses occurring during polymerization at the inlay-tooth tissue interface. In some cases, the occurrence of hydroscopic compressive stress could support retention of the reconstruction. The hydroscopic expansion stress, in all cases, reaches insignificant values and, therefore, should not have a negative impact on the reconstructed tooth.

Our findings suggest that using a thicker cement layer does not necessarily have a detrimental effect on contraction stress or the retention of prosthodontic restorations, despite previous reports that increasing the cement thickness from 100 to 300 μm leads to an increase in the stress magnitude [50]. These findings were also supported by a finite element study on stress generated by a luting resin during the cementation of ceramic and composite inlays [51,52,53]. Our photoelastic study reveals that even though 200 µm cement layers demonstrate twice the nominal stress values of the 25 µm layers, this stress is significantly reduced in a relatively short time (i.e., 2–3 days) due to water absorption. With further water absorption, they reach even lower values than those generated for 25 µm cement layers for both luting materials with different water absorption [47].

In the present work, some simplifications were made to obtain results that can be related to clinical conditions. For example, cylindrical samples made from CuZn alloy (lack of water absorbency) were used to mimic the inlay, epoxy resin plate mimic dentine. There is currently no suitable tool or adequate method that would allow to study the inlay restoration stress state and affection of water aging. It is important to underline that all popular finite element studies of stress in dental restorations use certain simplifications and assumptions, and that these limit the reproduction of clinical conditions. In addition, in finite element method (FEM) studies, it is impossible to determine how the stress state will change under the influence of water absorption [54,55]. The implementation of photoelastic tests to assess the state of stress at the interface between the dentine and the cement seems to have greater clinical value than FEM studies primarily because it also takes into account the conditions in the mouth (temperature and humidity).

These results are also of value when cementing prosthodontic crowns, bridges or posts and cores. The use of luting cements with higher water absorption values (self-adhesive cements) [19] may result in expansion stresses. This phenomenon will have a desirable effect, changing the direction (vectors) of the acting forces from contraction to compressive stresses, which may improve retention of prosthodontic restorations. Additionally, in clinical conditions, high compressive stresses should not be expected due to the elastic properties of tooth tissues: the dentin is an essential part of prepared prosthetic pillar, and can deform naturally under compressive stress [56,57].

## 5. Conclusions

Within the limitations of this study, it was found that thicker cement layers demonstrate higher contraction stresses. Moreover, applying a thin layer (approximately 25 µm) of the composite (NX3) and self-adhesive resin cements (Maxcem Elite Chroma) resulted in high hydroscopic expansion stresses (over ~6 MPa). The use of thicker layers (higher than 25 µm, but not exceeding 200 µm) may have a positive clinical effect, resulting in the creation of expansion stress that will potentially influence the sealing of the marginal gap and enhance inlay-tooth retention. The presented study simulated clinical conditions where the fit of an indirect restoration was not perfect and the thickness of the cement could vary within the internal fit of the indirect restoration.

## Figures and Tables

**Table 1:** The composition of resin cements.

Material	Type	Composition	Curing Time (s)	Manufacturer
NX3	Resin composite cement	TEGDMA, bis-GMA, fluoro-aluminosilicate glass (67.5% wt./47% vol.), activators, stabilizers, radiopaque agent	20	Kerr
Maxcem Elite Chroma	Self-adhesive resin cement	HEMA, GDM, UDMA, 1,1,3,3-tetramethylbutyl hydroperoxide TEGDMA, fluoro-aluminosilicate glass, GPDM, barium glass filler, fumed silica (69% wt.)	10	Kerr

TEGDMA—triethylene glycol dimethacrylate, bis-GMA—bisphenol A glycol dimethacrylate, HEMA—hydroxyethyl methacrylate, GDM—glycerol 1,3–dimethacrylate, UDMA—urethane dimethacrylate, GPDM—glycerol phosphate dimethacrylate.

**Table 2:** Relationship between the values of radial (σ_r_), circumferential (σ_θ_), and shrinkage stresses and the thickness of self-adhesive resin cement (Maxcem Elite Chroma) layers.

Cement Layer Thickness	σ_r_ MPa	σ_θ_ MPa	Shrinkage Stress MPa
the cement itself	6.6 ± 0.1	−7.9 ± 0.1	14.5 ± 0.1
approximately 25 µm	0.9 ± 0.1	−2.1 ± 0.2	3.1 ± 0.3
approximately 100 µm	2.8 ± 0.4	−4.2 ± 0.7	7.0 ± 0.5
approximately 200 µm	3.8 ± 0.2	−5.4 ± 0.3	9.2 ± 0.5
approximately 400 µm	3.8 ± 0.2	−5.4 ± 0.3	9.2 ± 0.5

**Table 3:** Relationship between the values of radial (σr), circumferential (σθ), and shrinkage stresses and the thickness of resin composite cement (NX3) layers.

Cement Layer Thickness	σ_r_ MPa	σ_θ_ MPa	Shrinkage Stress MPa
the cement itself	3.9 ± 0.4	−5.2 ± 0.5	9.1 ± 0.9
approximately 25 µm	0.7 ± 0.1	−1.8 ± 0.2	2.6 ± 0.3
approximately 100 µm	3.0 ± 0.2	−4.6 ± 0.2	7.6 ± 0.4
approximately 200 µm	3.9 ± 0.2	−5.4 ± 0.2	9.3 ± 0.4
approximately 400 µm	4.8 ± 0.2	−5.9 ± 0.3	10.7 ± 0.5

## Data Availability

The data presented in this study are available on request from the corresponding author.

## Clinical Significance

Differences in the thickness of the resin cement (higher than 25 µm, but not exceeding 200 µm) which might occur in inlay restoration, may have a positive clinical effect by compensating for contraction stress after a time depending on cement thickness and the composition of luting materials.
